# Expression of sterol regulatory element-binding transcription factor (SREBF) 2 and SREBF cleavage-activating protein (SCAP) in human atheroma and the association of their allelic variants with sudden cardiac death

**DOI:** 10.1186/1477-9560-6-17

**Published:** 2008-12-30

**Authors:** Yue-Mei Fan, Pekka J Karhunen, Mari Levula, Erkki Ilveskoski, Jussi Mikkelsson, Olli A Kajander, Otso Järvinen, Niku Oksala, Janita Thusberg, Mauno Vihinen, Juha-Pekka Salenius, Leena Kytömäki, Juhani T Soini, Reijo Laaksonen, Terho Lehtimäki

**Affiliations:** 1 Laboratory of Atherosclerosis Genetics, Department of Clinical Chemistry, Centre for Laboratory Medicine, Tampere University Hospital and Medical School, University of Tampere, Tampere, Finland; 2 Department of Forensic Medicine, Medical School, University of Tampere, Tampere, Finland; 3 Heart Centre, Department of Cardiac Surgery, Tampere University Hospital, Tampere, Finland; 4 Division of Vascular Surgery, Department of Surgery, Tampere University Hospital, Tampere, Finland; 5 Institute of Medical Technology, University of Tampere, Tampere, Finland; 6 Research Unit, Tampere University Hospital, Tampere, Finland; 7 Turku Centre for Biotechnology, University of Turku and Åbo Akademi University, Turku, Finland

## Abstract

**Background:**

Disturbed cellular cholesterol homeostasis may lead to accumulation of cholesterol in human atheroma plaques. Cellular cholesterol homeostasis is controlled by the sterol regulatory element-binding transcription factor 2 (SREBF-2) and the SREBF cleavage-activating protein (SCAP). We investigated whole genome expression in a series of human atherosclerotic samples from different vascular territories and studied whether the non-synonymous coding variants in the interacting domains of two genes, *SREBF-2 *1784G>C (rs2228314) and *SCAP *2386A>G, are related to the progression of coronary atherosclerosis and the risk of pre-hospital sudden cardiac death (SCD).

**Methods:**

Whole genome expression profiling was completed in twenty vascular samples from carotid, aortic and femoral atherosclerotic plaques and six control samples from internal mammary arteries. Three hundred sudden pre-hospital deaths of middle-aged (33–69 years) Caucasian Finnish men were subjected to detailed autopsy in the Helsinki Sudden Death Study. Coronary narrowing and areas of coronary wall covered with fatty streaks or fibrotic, calcified or complicated lesions were measured and related to the *SREBF-2 *and *SCAP *genotypes.

**Results:**

Whole genome expression profiling showed a significant (p = 0.02) down-regulation of *SREBF-2 *in atherosclerotic carotid plaques (types IV-V), but not in the aorta or femoral arteries (p = NS for both), as compared with the histologically confirmed non-atherosclerotic tissues. In logistic regression analysis, a significant interaction between the *SREBF-2 *1784G>C and the *SCAP *2386A>G genotype was observed on the risk of SCD (p = 0.046). Men with the *SREBF-2 *C allele and the *SCAP *G allele had a significantly increased risk of SCD (OR 2.68, 95% CI 1.07–6.71), compared to *SCAP *AA homologous subjects carrying the *SREBF-2 *C allele. Furthermore, similar trends for having complicated lesions and for the occurrence of thrombosis were found, although the results were not statistically significant.

**Conclusion:**

The results suggest that the allelic variants (*SREBF-2 *1784G>C and *SCAP *2386A>G) in the cholesterol homeostasis regulating SREBF-SCAP pathway may contribute to SCD in early middle-aged men.

## Background

Cholesterol is an essential component of cellular membranes. Cellular cholesterol homeostasis is controlled by sterol regulatory element-binding transcription factors (SREBFs), which are transcription factors that modulate the transcription of genes involved in lipid and cholesterol metabolism. In sterol-depleted cells, SREBF cleavage activating protein (SCAP) forms a complex with SREBF and assists in its transportation to the Golgi apparatus where it can be processed by two specific proteases and then release the amino-terminal transcription-activation domain of the SREBF. The active form of SREBF can then travel to the nucleus where it binds to the promoters of target genes [1]. The SREBFs consist of three different SREBF isoforms, SREBF-1a, SREBF-1c and SREBF-2. They are produced by two genes, with SREBF-1a and -1c transcribed from a single gene by alternative promoter use and splicing, and SREBF-2 from a separate gene [2]. There is considerable evidence suggesting that SREBF-2 is closely associated with cholesterol metabolism, while SREBF-1 is more associated with the control of genes involved in fatty acid metabolism [3,4].

As regulatory factors of cellular cholesterol metabolism, SREBF-2 as well as SCAP together with low-density lipoprotein receptor and 3-hydroxy-3-methylglutaryl coenzyme A reductase are assumed to be down-regulated in human atherosclerotic plaques due to the cholesterol overload. However, we were able to find only one publication quantitatively comparing the expression of SREBF-2 mRNA in human atherosclerotic plaques with nearby macroscopically intact carotid artery tissue [5]. This analysis revealed no differences in the expression of low-density lipoprotein receptor, 3-hydroxy-3-methylglutaryl coenzyme A reductase or SREBF-2 between the plaque and the control tissues with no histological classification of atherosclerosis [5].

Given the central role of the SREBF-SCAP pathway in the regulation of cholesterol, the variation in the *SREBF-2 *and *SCAP *locus might affect the progression of atherosclerosis. SREBF-2 is encoded by a gene (sterol regulatory element binding transcription factor, *SREBF-2*) on human chromosome 22q13 [2], which is composed of 19 exons [6]. A common polymorphism is located in exon 10, namely the 1784G>C (rs2228314) transversion resulting in the substitution of a glycine by an alanine at amino acid 595 of the SREBF-2 protein. This polymorphism was reported to be associated with serum levels of total cholesterol in hypercholesterolemic subjects from Switzerland and Israel [7] and from northern China [8]. This polymorphism was also associated with the intima-media thickness in French asymptomatic men [9].

SCAP is a membrane-bound protein containing two distinct domains: a sterol-sensing domain which serves as a cell cholesterol sensor and a carboxy-terminal domain with multiple WD repeats which mediates protein-protein interaction [10]. The human *SCAP *gene is located at chromosome 3p21.3 with 23 exons [11]. The *SCAP *gene has an exonic polymorphism located at exon 16 (A-to-G transition, 2386A>G) and leading to isoleucine-to-valine substitution (I796V) [12]. This polymorphism has been reported to be a significant predictor of the response of total cholesterol and triglyceride levels to simvastatin treatment [13], but not to fluvastatin [14] and pravastatin therapy [15].

There are two publications about the interaction effect of *SREBF-2 *1784G>C and *SCAP *2386A>G at present. The combination of *SREBF-2 *1784G>C and *SCAP *2386A>G has an effect on LDL cholesterol levels among familial hypercholesterolemia females [16]. Interestingly, the *SCAP *2386A>G genotypes were found to modify the association between *SREBF-2 *1784G>C and myocardial infarction (MI) in men [17].

Therefore, we investigated the expression of *SREBF-2 *and *SCAP *in human atherosclerotic tissues and compared them in different arterial beds, i.e. the carotid, aorta and femoral region, to methodologically accurately confirmed normal arterial tissue. We also related the variations 1784G>C and 2386A>G in the interacting domains of the *SREBF-2 *and *SCAP *genes to the areas of the different types of atherosclerotic lesions in the coronary arteries and to the risk of sudden cardiac death (SCD) and acute myocardial infarction (AMI) in an autopsy series of 300 Finnish men included in the Helsinki Sudden Death Study (HSDS).

## Methods

### Vascular samples

The vascular sample series consists of 20 samples from the carotid artery, the femoral artery and the abdominal aorta (15 males, 5 females, aged 69 ± 11 years). Six control samples were taken from internal mammary arteries (4 males, 2 females, aged 66 ± 11 years). The study was approved by the Ethics Committee of Tampere University Hospital, and the study subjects gave their informed consent. The samples were taken from patients subjected to open vascular surgical procedures at the Division of Vascular Surgery, Tampere University Hospital. The vascular samples were histologically classified according to the recommendations of the American Heart Association [18].

### RNA isolation and genome-wide expression analysis

The fresh tissue samples were soaked in RNALater solution (Ambion Inc., Austin, TX, USA) and isolated with Trizol reagent (Intitrogen, Carlsbad, CA, USA) and the RNAEasy Kit (Qiagen, Valencia, CA, USA). The concentration and quality of RNA were evaluated spectrophotometrically with Agilent (BioPhotometer, Eppendorf, Wesseling-Berzdorf, Germany). Over 23,000 known genes and gene candidates were analyzed using Sentrix Human-8 Expression BeadChips, according to manufacturer's instructions (Illumina, San Diego, CA, USA). In brief, 200 ng aliquot of total RNA from each sample was amplified to cDNA. In vitro transcription reaction of cDNA to cRNA was performed overnight (14 h) including biotin-11-dUTP for labeling the cRNA product. Each sample cRNA (1500 ng) was hybridized to Illumina's Sentrix Human-8 Expression BeadChip arrays. Hybridized biotinylated cRNA was detected with 1 μg/ml Cyanine3-streptavidine (Amersham Biosciences, Pistacataway, NJ, USA). BeadChips were scanned with the Illumina BeadArray Reader. The method has been described in more detail previously [19].

### Bioinformatics analysis of homology recognition of protein sequences and two SNPs

Sequence homologues for the SCAP and SREBF-2 proteins were obtained by PSI-BLAST [20]. The bioinformatics analysis of the mutations was performed at the sequence level as described by Thusberg et al. [21]. The secondary structures were predicted by the programmes PSIPRED [22] and PHD [23].

### Helsinki Sudden Death Autopsy Study

The HSDS was launched to study the lifestyle and genetic factors predisposing to sudden death in Finnish middle-aged men who lived in Helsinki and its surroundings. The present autopsy series was collected during 1991–1992 (n = 300) at the Department of Forensic Medicine, University of Helsinki. The cause of death was cardiac in 39% (n = 117), other diseases in 21% (n = 63) and suicides or accidents in 40% (n = 120) of the subjects. The study was approved by the Ethics Committee of the Department of Forensic Medicine, University of Helsinki.

### Measurement of the area of atherosclerosis by computer-assisted morphometry

At autopsy, the proximal parts of the left anterior descending coronary artery (LAD), right coronary artery (RCA) and left circumflex coronary artery (LCX) were collected for analysis. The definition of atherosclerotic lesions was based on the protocols of two international studies: the International Atherosclerosis Project, Standard Operating Protocol 1962 [24] and the WHO Study Group in Europe [25]. The areas of coronary artery wall covered by fatty streaks as well as fibrous and complicated lesions were measured with a computer-assisted planimetric technique. The details have been described in a previous publication [26]. The areas of coronary artery calcification were measured by examining the radiograph taken from the dissected coronary arteries. The mean proportional area of each particular atherosclerotic change in the three coronary arteries was used for statistical analysis. Of the series of 300 men, arterial samples for the measurements of atherosclerotic changes were available from 290 men for the analysis of three coronary arteries.

### Measuring the percentage of stenosis in silicone rubber casts of the coronary arteries

At autopsy, coronary angiography was performed using vulcanising liquid silicone rubber [27]. The proximal, middle and distal stenosis of the main trunks of the three main epicardial coronary arteries (LAD, LCX and RCA) were measured from the rubber cast model. The stenosis percentage was obtained by dividing the diameter (millimetres) of the greatest stenosis by the diameter of the nearest proximal undamaged part of the cast model of the artery, resulting in nine measurements of the degree of stenosis for each individual. The most severe stenosis was used to define the extent of coronary narrowing for each individual. These measurements were available for 279 men.

### Determining the MI phenotype at autopsy

Coronary thrombosis and myocardial infarction were recorded at autopsy, and the presence of MI was confirmed by nitro blue tetrazolium staining and by a histological examination of the myocardium. The presence of neutrophil granulocytes was considered diagnostic of an AMI. Thrombosis was defined by a reddish clot attached to the coronary wall if the clot could not be detached with saline flushing.

### Collection of risk factor data

A spouse, a relative or a close friend of the deceased could be interviewed in 147 cases. The questionnaire included a review of risk factors including hypertension, diabetes, past and recent smoking, drinking habits and previous illnesses. On the basis of these interviews, men were classified as smokers or non-smokers. Ex-smokers were included in the category of smokers for statistical analysis. Average daily alcohol consumption of the deceased was calculated based on the information given by the persons interviewed. Based on questions concerning previous illnesses, 50 men had suffered from hypertension and 22 from diabetes.

### DNA extraction and genotyping

DNA was extracted from frozen cardiac muscle samples. The *SCAP *2386A>G genotyping was based on PCR amplification, restriction enzyme analysis and DNA electrophoresis. The DNA samples were amplified by PCR, using the primers 5'-TTGTGCTGCGCGGCCACCTCA-3' and 5'-AGGAGGAAAGGGCAGCCGCAC-3'. PCR was performed in a volume of 50 μl. Cycle conditions were 94°C for 4 min, then 28 cycles of 94°C for 1 min, 64°C for 1 min and 72°C for 1 min, with a final extension step of 5 min at 72°C in a PTC-225 thermal cycler (MJ research, Massachusetts, USA). 10% DMSO was included in PCR reaction. The PCR-amplified DNA fragment was incubated for 16 hours with 10 U of *MslI *(New England Biolabs). Electrophoresis was performed on a 3% agarose gel containing ethidium bromide. Genotype information was obtained for 269 men.

For the *SREBF-2 *1784G>C genotyping, DNA samples were genotyped by employing the 5' nuclease assay for allelic discrimination, with the ABI Prism 7000 Sequence Detection System (Applied Biosystems, Foster City, CA). PCR reaction containing genomic DNA, 2 × TaqMan universal PCR Master Mix, 900 nM of each primer and 200 nM of each probe was performed in 96-well plates according to standard protocol in a total volume of 25 μl. After cycling, end-point fluorescence was measured, and genotype calling was carried out by the allelic discrimination analysis module. Genotyping was successful in 271 men. As a means of quality control for genotyping, empty controls and random duplicate samples were used.

### Statistical analysis

The non-parametric Mann-Whitney U test was used for comparison of gene expression between atherosclerotic and control tissues. Complete data on *SREBF-2 *and *SCAP *genotypes as well as autopsy data were available in 247 cases; this autopsy cohort constituted the final study population. Data analysis for continuous variables was based on analysis of variance (ANOVA) and analysis of covariance (ANCOVA), in which the possible confounding effects of age, body mass index (BMI) and hypertension were taken into account by including them in the model as covariates. Categorical variables were compared with the χ^2 ^test. Fisher's exact test was used to test the genotype frequencies under Hardy-Weinberg equilibrium. Non-normally distributed data concerning the areas of atherosclerotic changes were analysed after square-root transformation, but the results are displayed as crude data. We analysed the interaction between *SREBF-2 *1784G>C and *SCAP *2386A>G genotypes using logistic regression. Logistic regression analysis was also used to determine odds ratios for SCD according to *SREBF-2 *1784G>C and *SCAP *2386A>G genotype groups. The statistical analysis was performed by means of SPSS Version 14.0.1 (SPSS Inc., Chicago, USA). The level of significance was set at p < 0.05.

## Results

### *SREBF-2 *and *SCAP *expression in atherosclerotic tissue

The *SREBF-2 *gene expression was reduced 1.5-, 1.1- and 1.3-fold in the carotid artery (n = 9), aorta (n = 7) and femoral artery (n = 4), respectively. Only the *SREBF-2 *expression level in the carotid artery was significantly lower in atherosclerotic tissue than in control tissue (n = 6, p = 0.02 by Mann-Whitney U test). The similar trend of the *SCAP *gene expression was also found in three arteries, although it did not reach statistically significant. When all atherosclerotic arterial samples (type IV-V plaques) were pooled, there were no differences in the mean gene expression of either *SREBF-2 *or *SCAP *in atherosclerotic tissue as compared with histologically confirmed healthy control samples (type 0).

### Bioinformatics analysis

The search by PSI-BLAST yielded 64 homologue sequences for SCAP and 62 sequences for SREBF-2, after the removal of duplicates and hypothetical proteins.

The mutation position in SCAP is physicochemically conserved (the conserved property is hydrophobicity), and the frequencies of the physiochemically similar amino acids (V, L and I) are approximately equal. The disease-causing mutation studied here (I796V) is thus a conservative substitution. The mutation position in SREBF-2 is not at all conserved, and the following amino acids are found at this position: Y, V, T, S, R, Q, L, K, G, E and A. Q and E are the amino acids with the highest frequency of occurrence in the alignment. The mutations do not seem to have a direct structural effect on the proteins.

### Characteristics of *SREBF-2 *and *SCAP *genotypes in study subjects and their association with the area of different atherosclerotic changes

Of all the 247 men with genotype and autopsy data available, the allele frequencies did not differ significantly between the subpopulations with or without interview data. In addition to the *SREBF-2 *and *SCAP *genotypes as well as autopsy data, full risk factor data was available in 124 cases. The distributions of *SREBF-2 *1784G>C and *SCAP *2386A>G genotypes were in accordance with the Hardy-Weinberg equilibrium (Fisher's exact test p = 0.22 and p = 0.06, respectively). The characteristics of the study subjects according to *SREBF-2 *and *SCAP *genotype are shown in Table 1. There were no differences in any characteristics according to *SREBF-2 *genotype. Only *SCAP *2386A>G GG carriers had a higher prevalence of hypertension than subjects with other genotypes. No significant differences existed in the occurrence of SCD, AMI and thrombosis between the *SREBF-2 *or *SCAP *genotypes (Table 1).

**Table 1:** Characteristics of the study subjects by *SCAP *and *SREBF-2 *genotype

		*SCAP*				*SREBF-2*		
	AA	GA	GG	P value	GG	GC	CC	P value
Number of subjects	85	132	30		140	97	10	
Age (years)	51.9 ± 9.5	51.6 ± 9.4	55.6 ± 10.3	0.114	51.9 ± 9.3	52.9 ± 9.9	49.1 ± 11.0	0.445
BMI (kg/m^2^)	25.4 ± 5.1	25.4 ± 4.9	24.3 ± 4.1	0.497	25.2 ± 4.9	25.3 ± 4.7	25.1 ± 6.3	0.979
Alcohol consumption (g/day)	101.5 ± 116.4	106.5 ± 106.6	74.0 ± 68.2	0.549	110. 8 ± 110.9	91.9 ± 100.4	42.7 ± 84.2	0.340
Smoking	35 (72.9)	64 (80.0)	13 (92.9)	0.256	62 (82.7)	45 (72.6)	5 (100.0)	0.177
Hypertension	6 (13.6)	31 (46.3)	5 (38.5)	0.002	23 (32.9)	18 (36.0)	1 (25.0)	0.872
Diabetes	6 (13.6)	11 (16.4)	2 (15.4)	0.924	13 (18.6)	6 (12.0)	0 (0.0)	0.423
AMI	7 (8.2)	17 (12.9)	4 (13.3)	0.537	13 (9.3)	14 (14.4)	1 (10.0)	0.466
Thrombus	4 (4.7)	14 (10.6)	2 (6.7)	0.285	10 (7.1)	9 (9.3)	1 (10.0)	0.818
SCD	29 (34.1)	51 (38.6)	15 (50.0)	0.306	54 (38.6)	38 (39.2)	3 (30.0)	0.850

Values are mean ± SD or n (%). Abbreviations: AMI, acute myocardial infarction; BMI, body mass index; MI, myocardial infarction; SCD, sudden cardiac death; Analysis by ANOVA or χ^2 ^test

The association between the *SREBF-2 *1784G>C and *SCAP *2386A>G genotypes and the area of different atherosclerotic changes are shown in Table 2. We found borderline significant associations between the *SCAP *2386A>G genotypes and the mean percentage area of complicated lesions as well as between the *SREBF-2 *1784G>C genotypes and fatty streak area (Table 2). These results remained when ANCOVA was employed after adjustment for age, BMI and hypertension.

**Table 2:** Mean percent area of different types of atherosclerotic lesions in coronary arteries by *SCAP *and *SREBF-2 *genotype

		*SCAP*				*SREBF-2*		
	AA	GA	GG	P value	GG	GC	CC	P value
	85	132	30		140	97	10	
Fatty streaks	5.75 ± 4.87	6.12 ± 5.14	4.90 ± 4.19	0.463	5.51 ± 4.21	6.60 ± 5.88	3.17 ± 2.93	0.060
Fibrotic	3.06 ± 3.29	3.40 ± 3.77	2.95 ± 3.46	0.711	3.05 ± 3.44	3.61 ± 3.82	2.01 ± 2.45	0.268
Complicated	0.68 ± 1.95	1.64 ± 4.12	1.17 ± 2.38	0.053	1.26 ± 3.83	1.26 ± 2.66	1.10 ± 1.83	0.989
Calcification	2.08 ± 3.23	2.59 ± 4.97	3.06 ± 5.68	0.542	2.63 ± 4.73	2.38 ± 4.43	1.10 ± 2.46	0.572
Stenosis	36.99 ± 23.23	37.08 ± 21.12	38.36 ± 21.76	0.956	36.10 ± 22.26	39.14 ± 21.20	34.47 ± 22.92	0.550

Values are mean ± SD. Analysis by ANOVA.

### The interaction of *SREBF-2 *and *SCAP *genotypes on the risk of SCD

A significant interaction effect of *SREBF-2 *and *SCAP *genotypes was found on the risk of SCD (p = 0.046). The percentage of men with the *SREBF-2 *C allele in combination with the *SCAP *G allele among SCD victims tended to be higher than that found in the non-SCD group (34.7% vs. 26.3%). Carriage of the *SREBF-2 *C allele and the *SCAP *G allele was associated (OR 2.68, 95% confidence interval [CI] 1.07–6.71, p = 0.035) with an increased risk of SCD when compared with subjects carrying the *SREBF-2 *C allele and those with the *SCAP *AA genotype (Table 3). The significant difference remained after adjustment for age, BMI and hypertension.

**Table 3:** Joint association between *SCAP *and *SREBF-2 *genotypes and SCD risk

Genotype		Number of subjects		OR (95% CI)
*SCAP*	*SREBF-2*	SCD	Non-SCD	
AA	GC+CC	8	26	1.00 (Reference)
	GG	21	30	2.28 (0.86–5.99)
AG+GG	GC+CC	33	40	2.68 (1.07–6.71)
	GG	33	56	1.92 (0.78–4.72)

Abbreviation: OR, odds ratio; SCD, sudden cardiac death.

Furthermore, we found a similar trend suggesting that there were more carriers of the combination *SREBF-2 *C allele and *SCAP *G allele among men with AMI, complicated lesions and coronary thrombosis than among those who did not suffer from these ailments but here the results were not statistically significant (Table 4). Carriage of the *SREBF-2 *C allele and the *SCAP *G allele was associated (OR 2.4, p = 0.06) with an increased risk of having complicated lesions when compared with subjects carrying the *SREBF-2 *C allele and the *SCAP *AA genotype. The combination of the *SREBF-2 *C allele and the *SCAP *G allele was present in 8 of the 20 men (40%) with coronary thrombosis, whereas the *SREBF-2 *C allele in combination with the *SCAP *AA genotype was present in 2 of the 20 men (10%) with coronary thrombosis (Table 4). There were also more carriers of the *SREBF-2 *C allele in combination with the *SCAP *G allele among men with AMI than among men who had died of a non-AMI (Table 4).

**Table 4:** Distributions of *SCAP* and *SREBF-2* genotypes among men with SCD, AMI, thrombosis and complicated lesions (CL), and among those who had not suffered from the same at the time of death

Genotype		N (%)		N (%)		N (%)		N (%)	
*SCAP*	*SREBF-2*	SCD	Non-SCD	With CL	Without CL	Thrombus	Without thrombus	AMI	Non-AMI
AA	GC+CC	8 (8.4)	26 (17.1)	8 (8.8)	26 (16.7)	2 (10.0)	32 (14.1)	4 (14.3)	30 (13.7)
	GG	21 (22.1)	30 (19.7)	18 (19.8)	33 (21.2)	2 (10.0)	49 (21.6)	3 (10.7)	48 (21.9)
AG+GG	GC+CC	33 (34.7)	40 (26.3)	31 (34.1)	42 (26.9)	8 (40.0)	65 (28.6)	11 (39.3)	62 (28.3)
	GG	33 (34.7)	56 (36.8)	34 (37.4)	55 (35.3)	8 (40.0)	81 (35.7)	10 (35.7)	79 (36.1)

Values are n (%). Abbreviations: AMI, acute myocardial infarction; CL, complicated lesions; SCD, sudden cardiac death.

## Discussion

We showed that *SREBF-2 *expression in the carotid artery was decreased in atherosclerotic tissue as compared to histologically confirmed healthy control tissue. The similar trend of *SCAP *gene expression was also found, although there was no statistically significant difference between the atherosclerotic tissue and control samples. In the present study, the *SREBF-2 *1784G>C or *SCAP *2386A>G genotypes were also not associated with SCD alone. However, we found that the *SREBF-2 *1784G>C and *SCAP *2386A>G genotypes have a significant interaction on the risk of SCD. The combination of the *SREBF-2 *C allele and the *SCAP *G allele was associated with an increased risk of SCD when compared with subjects carrying the *SREBF-2 *C allele and the *SCAP *AA genotype. Our results also suggest that this association may be due to the presence of more complicated lesions and thrombosis among the subjects with the *SREBF-2 *C allele in combination with the *SCAP *G allele.

Serum cholesterol is a major component in the causal pathway of atherosclerosis, which has been verified in familial hypercholesterolemia patients [28]. The cellular cholesterol homeostasis is subject to a regulatory feedback system that senses the level of cholesterol in cellular membranes and modulates the transcription of genes encoding enzymes involved in cholesterol biosynthesis and in the uptake of plasma lipoproteins. As regulators of this cholesterol metabolism, *SREBF-2 *and *SCAP *might play a role in the progression of atherosclerosis. Our gene expression results with human tissues are in agreement with *in vivo *findings indicating that SREBF-2 is down-regulated by hypercholesterolemia in porcine aortas [29]. In another study, Forcheron et al. observed that the mRNA expression of SREBF-2 was unchanged in human atheroma when compared with nearby macroscopically intact tissue [5]. Although there was no histological evidence of atherosclerosis in the control vessels, however, the absence of atherosclerosis cannot be explicitly excluded, and thus it is therefore possible that the expression of SREBF-2 was already near-maximally suppressed, explaining the results of unaltered relative gene expression. Therefore, our results support the notion of the suppression of SREBF-2 in atherosclerotic lesions which are usually bathed in high levels of lipoproteins.

The *SREBF-2 *1784G>C variant is located in carboxyl-terminal regulating region, which forms a complex with the carboxyl-terminal WD repeat domain that contains *SCAP *2386A>G substitution. Therefore, the domains of *SREBF-2 *and *SCAP *that contain the *SREBF-2 *1784G>C and *SCAP *2386A>G variation directly interact with each other. A functional study of the *SREBF-2 *1784G>C variant has shown that the SREBF-2 CC isoform results in a decreased SREBF-2 cleavage-rate in vitro [7]. Moreover, this variant was associated with serum total cholesterol in hypercholesterolemic subjects [7,8], and carotid intima media thickness values were higher in homozygous subjects for the presence of the C allele [9]. However, no functional studies of the *SCAP *2386A>G variant have been carried out. There are only several studies with conflicting results regarding the influence of the *SCAP *2386A>G variant on lipid-lowering therapy [13-15].

Our results are in accordance with a previous study in which the *SREBF-2 *C allele in combination with the *SCAP *G allele was associated with an increased risk of MI in males [17]. Our results extend the existing data, since there is no previous evidence suggesting that the same allelic combination of the SREBF-SCAP pathway that alters the risk of MI is also associated with the risk of SCD. The interaction of the *SREBF-2 *1784G>C and the *SCAP *2386A>G variant on SCD risk observed in our study may be partially due to an impaired formation and/or decreased stability of the SCAP/SREBF-2 complex and to altered interaction function, therefore altering cholesterol metabolism in human cells. A possible explanation through bioinformatics analysis for these two variations is that there is a physical interaction between these amino acids (SCAP 796 and SREBF-2 595), and this interaction is prevented by the variations, impairing the formation of the SCAP/SREBF-2 complex. However, because 3D structures of these proteins are not available, this must be interpreted with caution. According to the secondary structure predictions, the 1784G>C position in SREBF-2 is in α-helix. Equally high confidence values were obtained for the 2386A>G position in SCAP, but for a β-strand. The mutant sites are therefore on the ordered secondary structural elements and possibly on the surface of the proteins, which is supported by many predictors when the amino acid substitutions are considered to be tolerated. However, our study is epidemiological in nature and still need for empirical in silico proof for the interaction.

The limitations of our study include the two distinct sample materials we used: The vascular samples collected from elective patients subjected to open vascular surgical procedures were used for the *SREBF-2 *and *SCAP *mRNA expression study, and the HSDS samples were used for the association study between *SREBF-2 *1784G>C as well as *SCAP *2386A>G and coronary atherosclerosis. The form of atherosclerosis in these two materials might be different, because they were collected from different vascular territories with different local haemodynamic conditions. Other limitations are related to the nature of autopsy studies: since the subjects had died suddenly and most of them had not seen a doctor nor had any blood samples taken prior to their death, we were unable to determine the lipid levels. We also did not measure the cholesterol and apolipoprotein levels in post-mortem samples. Furthermore, this study only enrolled Finnish men. We cannot ascertain whether the interactive effect of the *SREBF-2 *1784G>C and the *SCAP *2386A>G variant would extend to other populations or women, as well.

In summary, we demonstrated a down-regulated expression of *SREBF-2 *in atherosclerotic carotid plaques as compared with histologically confirmed normal tissues. We also showed a gene-gene interaction between the *SREBF-2 *1784G>C and the *SCAP *2386A>G genotypes on the risk of developing SCD in middle-aged men. We conclude that *SREBF-2 *and *SCAP *may be implicated in the progression of atherosclerosis and the risk of developing SCD. Future structure-function and replication studies with large samples are required to improve the understanding of how *SREBF-2 *and *SCAP *function, in addition to explaining the results presented here.

## Competing interests

The authors declare that they have no competing interests.

## Authors' contributions

YMF participated in the study design, performed the statistical analysis and drafted the manuscript. PJK participated in designing of the Helsinki Sudden Death Study (HSDS), in performing of autopsies and collecting of coronary samples, in performing of interviews of the next of kin, and revising the final draft of the manuscript. ML participated in vascular samples collection and isolation of RNA. EI, JM and OAK participated in the analysis and interpretation of the morphometric, coronary cast, the risk factor and the MI classification data of the HSDS. OJ and JPS performed surgical procedures for vascular samples. NO performed surgical procedures for vascular samples and revised the manuscript critically. JT and MV performed bioinformatics analysis. LK and JTS participated in executing of microarray lab work and pre-analyzing the results. RL participated in the study design. TL participated in the study design and revised the manuscript critically. All authors read and approved the final manuscript for publication.
